# GluA2 is rapidly edited at the Q/R site during neural differentiation *in vitro*

**DOI:** 10.3389/fncel.2015.00069

**Published:** 2015-03-05

**Authors:** Svenja Pachernegg, Yvonne Münster, Elke Muth-Köhne, Gloria Fuhrmann, Michael Hollmann

**Affiliations:** ^1^Department of Biochemistry I - Receptor Biochemistry, Ruhr University BochumBochum, Germany; ^2^International Graduate School of Neuroscience, Ruhr University BochumBochum, Germany; ^3^Ruhr University Research School, Ruhr University BochumBochum, Germany

**Keywords:** RNA editing, ESCs, NEPs, NSCs, ADAR2, editing assay, Q/R site, R/G site

## Abstract

The majority of AMPA receptors in the adult brain contain GluA2 subunits, which can be edited at the Q/R site, changing a glutamine to an arginine within the ion pore. Q/R editing renders AMPARs virtually Ca^2+^-impermeable, which is important for normal AMPA receptor function. Thus, all GluA2 subunits are Q/R-edited in the adult brain. However, it has remained controversial precisely when editing sets in during development. In the present study, we show that GluA2 mRNA is very rapidly Q/R-edited immediately after its appearance, which is after 4.5 days of differentiation from 46C embryonic stem cells (ESCs) to neuroepithelial precursor cells (NEPs). At this time point, most of the GluA2 transcripts were already edited, with only a small fraction remaining unedited, and half a day later all GluA2 transcripts were edited. This can be explained by the observation that the enzyme that Q/R-edits GluA2 transcripts, ADAR2, is already expressed in the cell well before GluA2 transcription starts, and later is not significantly upregulated any more. Editing at another site works differently: The R/G site within the ligand-binding domain was never completely edited at any of the developmental stages tested, and the enzyme that performs this editing, ADAR1, was significantly upregulated during neural differentiation. This confirms previous data suggesting that R/G editing, in contrast to Q/R editing, progresses gradually during development.

## 1. Introduction

The molecular diversity of ionotropic glutamate receptors (iGluRs) is large, since 18 different subunits have been discovered in mammals. These subunits are grouped into four different subfamilies, namely α-amino-3-hydroxy-5-methyl-4-isoaxole propionic acid receptors (AMPARs), kainate receptors (KARs), delta receptors, and N-methyl-D-aspartate receptors (NMDARs) (Mayer and Westbrook, 1987; Collingridge and Lester, 1989; Hollmann and Heinemann, 1994). Their molecular diversity is further increased by a number of post-transcriptional (RNA editing and splicing) and post-translational (e.g., N-glycosylation, phosphorylation, and palmitoylation) modifications. Both splicing and editing of AMPAR subunits impact their physiological properties.

All AMPAR subunits are spliced at the flip/flop splicing site, which leads to the expression of one of two 115 bp-long mutually exclusive sequences (Sommer et al., 1990). Flip splice variants activate faster and desensitize four times slower than flop splice variants (Sommer et al., 1990; Mosbacher et al., 1994; Koike et al., 2000), while flop variants show reduced current responses to glutamate than the flip variants (Sommer et al., 1990). Regarding the temporal expression of flip and flop splice variants, the flip variants predominate during early development, since the expression of flop splice variants is very low prior to P8 (Monyer et al., 1991).

Besides flip/flop splicing, the AMPAR subunits GluA2 through GluA4 undergo RNA editing at the R/G site; however, GluA2 is the only AMPAR which is edited at the Q/R editing site. During A-to-I editing, a genetically encoded adenosine in the pre-mRNA is converted into an inosine by oxidative deamination, and the inosine is then in turn read as a guanosine during translation (Benne et al., 1986). The AMPAR subunits GluA2 to GluA4 can undergo editing at the R/G site in the S2 domain of the receptor (Lomeli et al., 1994). R/G editing exchanges the genetically encoded arginine (R; encoded by AGA) at position 764 to a glycine (G; here encoded by the non-canonical triplet IGA, which is translated as GGA) (Lomeli et al., 1994). R/G-edited AMPARs desensitize faster and also recover faster from desensitization than unedited subunits (Lomeli et al., 1994). R/G editing gradually increases during neural development and upon neuronal cell maturation (Lomeli et al., 1994; Geiger et al., 1995; Orlandi et al., 2011). In adult rodent and human brains, roughly 50–85% of AMPARs are edited at the R/G site (Lomeli et al., 1994; Lai et al., 1997; Vollmar et al., 2004). More precisely, in the human hippocampus and neocortex, 55% and 60%, respectively, of AMPARs are R/G-edited (Vollmar et al., 2004).

In GluA2, Q/R editing leads to the exchange of the glutamine (Q; encoded by CAG) at the tip of the pore loop (position 607) to arginine (R; here encoded by the non-canonical triplet CIG, which is translated as CGG) (Sommer et al., 1991; Kuner et al., 2001). Since arginine is positively charged in contrast to the uncharged glutamine, one key feature of the ion pore is dramatically altered: Q/R-unedited GluA2 subunits are Ca^2+^-permeable, whereas Q/R-edited GluA2 subunits are virtually Ca^2+^-impermeable (Hollmann et al., 1991; Burnashev et al., 1992). Besides determining the Ca^2+^ permeability of AMPARs, Q/R editing of GluA2 also affects other electrophysiological properties. AMPARs with edited GluA2 show a linear current-voltage relationship, whereas AMPARs with unedited GluA2 have an inwardly rectifying current-voltage relationship (Bowie and Mayer, 1995; Geiger et al., 1995; Kamboj et al., 1995; Koh et al., 1995; Swanson et al., 1997). Furthermore, the single channel conductance of edited GluA2 is lower than that of unedited GluA2 (Swanson et al., 1997).

The majority of AMPARs *in vivo* are GluA1/GluA2 or GluA2/GluA3 heterotetramers (Geiger et al., 1995; Wenthold et al., 1996; Tsuzuki et al., 2001; Sans et al., 2003), and GluA2 dominates the receptor complex to the effect that the Ca^2+^ permeability and other electrophysiological properties of AMPARs depend on the Q/R editing state of the incorporated GluA2 subunit (Burnashev et al., 1992). In the adult rodent and human brain, virtually all GluA2 subunits are edited at the Q/R site (Paschen and Djuricic, 1995; Carlson et al., 2000; Kawahara et al., 2003; Jacobs et al., 2009; Barbon et al., 2010), thus, the majority of AMPARs is virtually Ca^2+^-impermeable. The extent to which GluA2 is edited at the Q/R site during embryonic and postnatal development is still a matter of debate and results obtained by several research groups differ. GluA2 was found to be almost fully edited at E15 in mice (Jacobs et al., 2009; Wahlstedt et al., 2009) and at P7 (the earliest time point examined in that particular study) in rats (Longone et al., 1998), but small amounts of unedited GluA2 subunits were found between E14 and P1 in rat neocortical organotypic slices as well (Hamad et al., 2011). One study found approximately 60% of GluA2 to be unedited at the Q/R site in human neural progenitor cells (Whitney et al., 2008). In human fetal brains, however, GluA2 was shown to be already fully edited at the Q/R site (Kawahara et al., 2004; Shtrichman et al., 2012). Furthermore, GluA2 was found to be completely edited prior to neuronal differentiation in human NT2 cells (Barbon et al., 2003). By contrast, in another study in human NT2 cells, GluA2 was completely edited at the Q/R site only after 3 weeks of retinoic acid (RA) treatment to induce neuronal differentiation (Lai et al., 1997). Interestingly, it was suggested that Q/R-unedited GluA2 does not play any important role during neural development at all, since GluA2^*R*/*R*^ mice, which are genetically edited at the Q/R site, show no differences in brain architecture (Kask et al., 1998).

RNA editing is mediated by the family of adenosine deaminases that act on RNA (ADARs). To date, three members of this family have been identified in rodents, namely ADAR1 (O'Connell et al., 1995), ADAR2 (Melcher et al., 1996b), and ADAR3 (Melcher et al., 1996a). Most of the editing targets for ADAR1 and ADAR2 encode for ion channels involved in neurotransmission, e.g., potassium channels (K_V_1.1; Bhalla et al., 2004), serotonin receptors (5-HT-2CR; Burns et al., 1997), γ-aminobutyric acid (GABA) receptors (GABA_A_; Rula et al., 2008), and iGluRs (GluA2 to GluA4; GluK1 and GluK2; Sommer et al., 1991; Lomeli et al., 1994). In the case of AMPARs, ADAR1 almost exclusively edits the R/G editing site, whereas ADAR2 edits both the R/G and Q/R editing sites (Wong et al., 2001).

**Figure 1:**
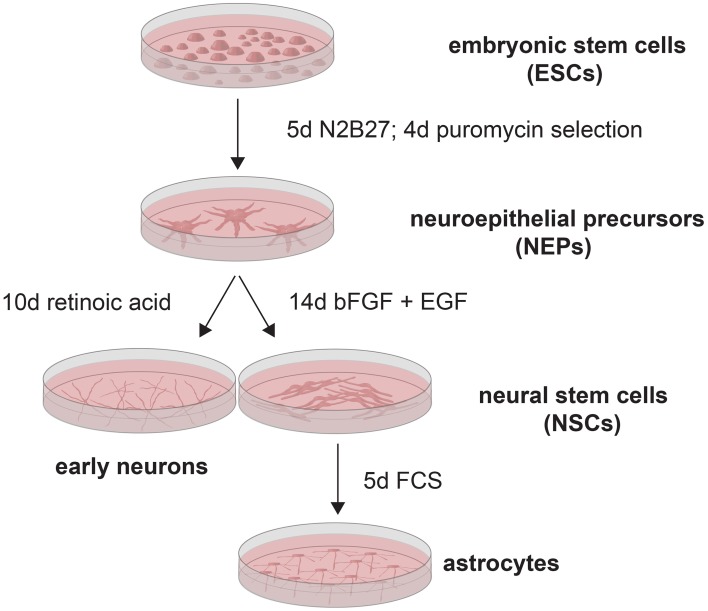
**46C ESCs (Aubert et al., 2003; Ying et al., 2003) were differentiated into NEPs via the prolonged cultivation in N2B27**. Subsequently, NEPs were selected by treatment with puromycin. NEPs were then differentiated either into early neurons by addition of retinoic acid, or into NSCs by addition of bFGF and EGF. Astrocytes were generated from NSCs by treatment with FCS.

Since the exact onset of Q/R editing of GluA2 during neural development has remained unclear thus far, and since one study found approximately 60% Q/R-unedited GluA2 in human neural progenitor cells (Whitney et al., 2008), we investigated the expression of GluA2 and its editing enzyme ADAR2 as well as the editing state of AMPARs during neural development in detail. For this, we used the genetically engineered 46C embryonic stem cell (ESC) line (Figure 1), which was generated by subcloning the CDS of EGFP as well as a puromycin resistance gene under control of the Sox1 promoter into E14Tg2a.IV cells (Aubert et al., 2003; Ying et al., 2003). Since Sox1 is the earliest known neuroectodermal marker (Wood and Episkopou, 1999), the cells show green fluorescence as soon as they are differentiated into Sox1-positive neuroepithelial precursor cells (NEPs). Subsequently, NEPs can be differentiated either into neurons via the treatment with retinoic acid (RA), or into radial glia-like neural stem cells (NSCs) via the prolonged cultivation in the neuroinductive medium N2B27 and the addition of basic fibroblast growth factor (bFGF) and epidermal growth factor (EGF) (Conti et al., 2005; Pollard et al., 2006; Muth-Köhne et al., 2010a). 46C NSCs can then be differentiated into astrocytes via the addition of fetal calf serum (FCS) (Glaser et al., 2007; Muth-Köhne et al., 2010a). We and others have shown that 46C ESCs and 46C-derived cells express appropriate stem cell and differentiation markers (Ying et al., 2003; Conti et al., 2005; Pollard et al., 2006; Glaser et al., 2007; Muth-Köhne et al., 2010a; Pachernegg et al., 2013). In this study, we show that ADAR2 is expressed both at the mRNA and protein levels prior to GluA2. Moreover, GluA2 mRNA is fully edited at the Q/R site before its protein expression sets in in early neurons.

## 2. Materials and methods

### 2.1. Cell culture

All 46C cell cultures were maintained at 37°C and 5% CO_2_. 46C ESCs were cultured in GMEM containing 10% FCS, 10% tryptose phosphate, 0.1 mM 2-mercaptoethanol, 1.8 mM glutamine, and 1000 U/ml leukemia inhibitory factor (LIF; Millipore). NEPs were differentiated from 46C ESCs by incubation in a neuroinductive medium (N2B27) as described previously (Ying et al., 2003). NSCs were differentiated from NEPs by prolonged cultivation in N2B27 medium and the addition of EGF and bFGF (both 10 ng/ml; Preprotech) (Conti et al., 2005). NEPs were differentiated into neurons via treatment with RA (10 μM) for 10 days, and NSCs were differentiated into astrocytes by adding 5% FCS to the medium for 14 days. Cells were allowed to grow for 2 days before RNA or protein isolation.

### 2.2. Reverse transcription and quantitative real time PCR

Total RNA was isolated from cell cultures and control tissue (mouse whole brain from postnatal day 3; P3) using the GenElute Mammalian Total RNA Mini-Kit (Sigma-Aldrich) according to the manufacturer's protocol. The total RNA isolation procedure included a DNase I (Promega) treatment. Per sample, 2 μg of RNA was used in reverse transcription reactions (SuperScriptII Reverse Transcriptase; Invitrogen) to generate cDNA. The cDNA was primed from random hexamer primers (Roche Diagnostics). In addition, a negative control was included, in which the amount of total RNA added to the other reactions was substituted by ddH_2_0. Prior to the quantitative real time PCRs (qRT-PCRs), every cDNA sample (and the negative control) was subjected to a PCR (Taq Polymerase; Invitrogen) and subsequent agarose gel analysis to control for possible genomic DNA contamination. Subsequently, 50 ng of cDNA per sample were used as template in qRT-PCRs on the Roche Light Cycler 2.0 using the FastStart DNA Master^*PLUS*^ SYBR Green I Kit (both Roche Diagnostics) according to the manufacturer's instructions. The reaction was performed with an initial pre-incubation for 10 min at 95°C, followed by 40 amplification cycles (95°C for 10 s, 59°C for 10 s, and 72°C for 20 s). A melting cycle was performed to determine the melting temperatures of the amplified products and to confirm their specificity (ramping at maximal speed to 95°C, immediately cooling down to 65°C for 15 s, and melting by increasing the temperature to 95°C at a rate of 0.1°C/s). Whole brain RNA of neonatal (P3) C57BL/6 mice served as positive control; brains were removed from mice in accordance with the Ruhr University animal treatment guidelines. For primer sequences, see Table 1. qRT-PCR results were analyzed using Roche LightCycler Software 3.5 (Roche Diagnostics). Quantitative real time data was obtained by mathematical modeling as described previously (Pfaffl, 2001; Muth-Köhne et al., 2010a,b; Pachernegg et al., 2013). The expression levels of the genes of interest were normalized to the expression levels of the housekeeping genes β-actin, GAPDH, and ubiquitin (2^Δ*Ct*^ method). To additionally compare the expression of genes in different 46C cell types to their expression in a control, the data was also normalized to the expression of genes in mouse whole brain (2^ΔΔ*Ct*^ method). Statistics were calculated using Prism 5.0 software (GraphPad).

**Table 1:** **Primers used in PCRs and qRT-PCRs**.

**Gen**	**Sequence 5′→3′**
GluA1	accatgaatgagtacattgagc
	tcgctacgggatttgtagc
GluA2 (R/G)	atgaatgagtacatcgagcag
	caaagccaccagcattgc
GluA2 (Q/R)	cctggtcagcagatttagcc
	cgacaccatcctctctacag
	ccctttggatttcctgactc (antisense-2)
GluA3	accatgaatgagtacattgagc
	tgcccgtgatttgtaacag
GluA4	atgaatgaatacattgagcagc
	caaagccaccagcattgc
ADAR1	agactctcaccagctgac
	atgtctcgcaggcttttctt
ADAR2-long	agactctcaccagctgac
	agaagtagatgggttccacg
ADAR2-short	gtgctagaggaaccagcag
	agaagtagatgggttccacg
β-actin	cgttgacatccgtaaagacct
	caaagccatgccaatgttgtctct
GAPDH	catcaacgaccccttcatt
	ctccacgacatactcagcac
ubiquitin	ctgggcggttgcttt
	ggttgactccttctggatgtt

All primer pairs span at least one exon-exon junction and were designed by using Primer3 (http://primer3.ut.ee/) with similar primer efficiencies (ranging between 89 and 96%), annealing temperatures, product lengths, and GC contents.

### 2.3. Editing assays

**Figure 2:**
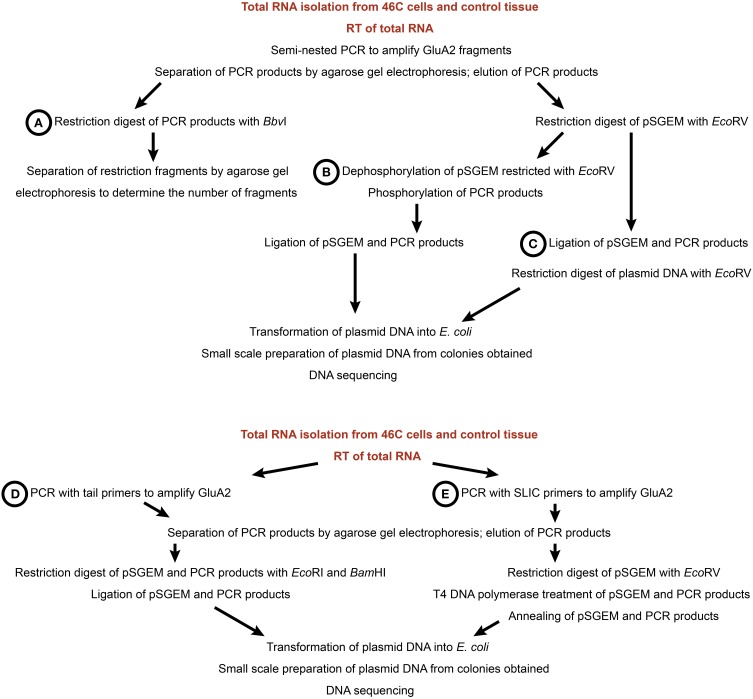
**Workflow of five different editing assay approaches**. **(A)** Restriction digest with *Bbv*I. **(B)** Blunt end cloning. **(C)** Ligation with subsequent restriction. **(D)** Sticky end cloning. **(E)** SLIC cloning. For all assays, total RNA was isolated from 46C cells and control tissue (mouse whole brain P3) and used in reverse transcriptions to generate cDNA (see Materials and Methods).

To analyze the editing state of AMPARs, five different editing assay approaches were tested (Figure 2). Since this study focused primarily on the Q/R editing of GluA2, the assays were mainly tested with this subunit. For all approaches, 50–100 ng cDNA were used as a template in PCRs (Phusion Polymerase, Promega) to amplify the AMPAR fragment which contains the editing site in question. For all editing assays except for those involving sticky end and SLIC cloning (see below), semi-nested PCRs were performed to increase the specificity of the PCR fragments. The GluA2 primers for semi-nested PCRs were based on the study by Whitney et al. (2008). For primer sequences, see Table 1. The 5 different editing assay approaches are described in the following.

#### 2.3.1. Restriction Digest with BbvI

Figure 2A This approach is based on the observation that edited and unedited GluA2 yield different restriction fragment patterns upon restriction digest with *Bbv*I (Whitney et al., 2008). In Q/R-edited GluA2, one of the *Bbv*I restriction sites is lost, yielding two DNA fragments (225 bp and 46 bp, respectively) after a restriction with *Bbv*I. By contrast, in Q/R-unedited GluA2 cDNA, three DNA fragments (145 bp, 80 bp, and 46 bp) are obtained after a restriction digest with *Bbv*I. Following the restriction digest, the DNA fragments were separated by agarose (2% w/v) gel electrophoresis.

In the other editing assays, the PCR products were subcloned into the pSGEM vector, which was then transformed into *E. coli* bacteria (heat shock transformation), individual clones were picked, and the plasmid DNA was then isolated from bacteria and sequenced (capillary sequencer Genetic Analyzer 3130xl; Applied Biosystems). The DNA sequencing-based editing assay approaches differed in the way the PCR products were subcloned into the pSGEM plasmid.

#### 2.3.2. Blunt end cloning

Figure 2B The pSGEM vector was cut with *Eco*RV (New England Biolabs) to generate blunt ends and was subsequently dephosphorylated by treatment with Antarctic Phosphatase (New England Biolabs). The PCR fragments were then phosphorylated by treatment with polynucleotide kinase (Promega). For the ligation reaction, plasmid and DNA inserts were incubated with 2.5 U T4 ligase (Fermentas) at 16°C over night.

#### 2.3.3. Ligation with Subsequent Restriction

Figure 2C After generating blunt-ended PCR fragments and *Eco*RV-cut (New England Biolabs) plasmid DNA, the dephosphorylation of the vector DNA as well as the phosphorylation of the PCR fragments was skipped. Plasmid DNA and PCR fragments were directly ligated and the ligation product was then cut with *Eco*RV (New England Biolabs) to linearize re-ligated plasmid DNA that did not incorporate a PCR fragment.

#### 2.3.4. Sticky end Cloning

Figure 2D Tail primers were used to insert restriction sites (*Eco*RI and *Bam*HI) at the 5′ and 3′ end of the PCR products, respectively. Both the DNA fragments and the plasmid DNA were then digested with *Eco*RI and *Bam*HI (New England Biolabs) and ligated.

#### 2.3.5. SLIC

Figure 2E For Sequence and Ligation Independent Cloning (SLIC; Li and Elledge, 2008), SLIC primers were used which add 16 bp to the PCR products; this overhang is homologous to the vector sequence adjacent to the *Eco*RV restriction site. The plasmid DNA was then linearized with *Eco*RV (New England Biolabs), and both plasmid and PCR fragments were treated with T4 DNA polymerase (Promega) to generate 5′ overhangs. Subsequently, vector and insert DNAs were annealed for 30 min at 37°C and the recombination intermediate was then transformed into *E. coli* bacteria (heat shock transformation).

### 2.4. Western blotting

Total membrane proteins of cell cultures were prepared by hypotonic lysis (10 mM HEPES/KOH, 1.5 mM KCl, 10 mM MgCl2, 0.5 mM DTT) followed by ultracentrifugation to pellet membrane-bound proteins (100,000 g; 1 h). For SDS-PAGE, 50 μg of membrane proteins were loaded per sample. After transferring the proteins to nitrocellulose membranes, the membranes were reversibly stained with Ponceau S (0.2% Ponceau S, 3% trichloroacetic acid, 3% sulfosalicylic acid) to check for the proper transfer of proteins onto the membrane. For immunoblot analysis, the following primary antibodies were used: anti-calnexin anti-ADAR2 (1:500; Santa Cruz Biotechnology) and anti-GluA2 (1:500; BD Biosciences). For detection, appropriate HRP-conjugated secondary antibodies (1:10,000; Sigma) were used. Whole brain proteins of neonatal (P3) C57BL/6 mice served as positive control. For the removal of antibodies from previously probed Western blots to allow reprobing of the membranes with another antibody, the membranes were incubated for 2 h in stripping buffer (25 mM glycine; pH 2.0, 1% SDS).

## 3. Results

### 3.1. Expression of GluA2 and ADARs in 46C cells

In this study, the mRNA expression of GluA2 in 46C ESCs, NEPs, NSCs, neurons, and astrocytes (Muth-Köhne et al., 2010a,b; Pachernegg et al., 2013) was verified via qRT-PCRs. Moreover, the mRNA expression of the editing enzymes ADAR1 and ADAR2 was also investigated in 46C cells. For exact values, see Table 3.

**Figure 3:**
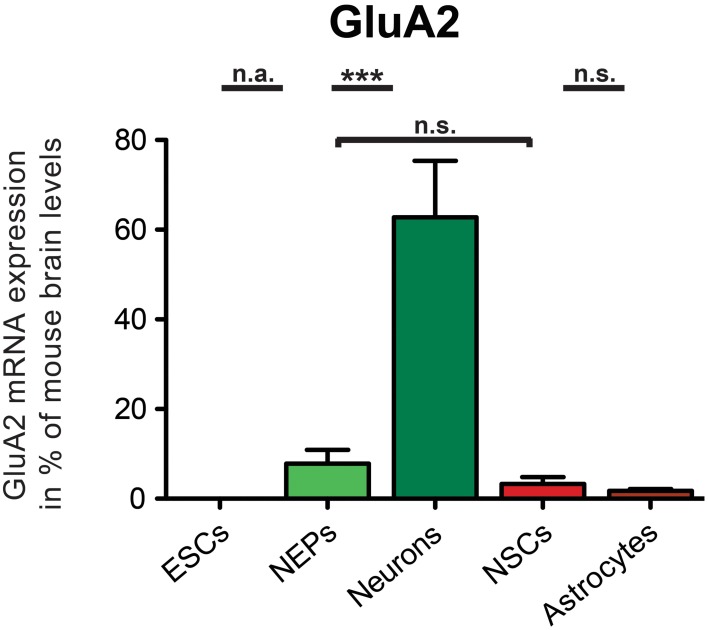
**Expression of GluA2 mRNA in 46C cells**. The mRNA expression of GluA2 was normalized to its expression in mouse whole brain P3 (2^ΔΔ*Ct*^). GluA2 transcripts were not expressed in 46C ESCs, but in NEPs, NSCs, neurons, and astrocytes. Its mRNA expression was significantly upregulated when 46C NEPs were differentiated into early neurons. Data represent means + SEM; statistical significances were assigned by One-Way ANOVA followed by Tukey's multiple comparison test; ***p < 0.001; n.s. = not significant; n.a. = not assigned (if the expression in at least one cell type was 0). n = 8-15 independent experiments.

GluA2 mRNA was not expressed in undifferentiated 46C ESCs, as has been found previously (Pachernegg et al., 2013) (Figure 3). By contrast, 46C NEPs robustly expressed GluA2 at the mRNA level (7.8% compared to its expression in mouse whole brain), and its expression was highly significantly upregulated (*p* < 0.001; Figure 3) upon neuronal differentiation from 46C NEPs into neurons. In 46C neurons, the mRNA expression of GluA2 accounted for 62.7% of its expression in mouse whole brain. GluA2 transcripts were slightly downregulated upon differentiation from 46C NEPs into NSCs and from 46C NSCs into astrocytes, respectively, although these differences in GluA2 mRNA expression were not statistically significant (Figure 3).

**Figure 4:**
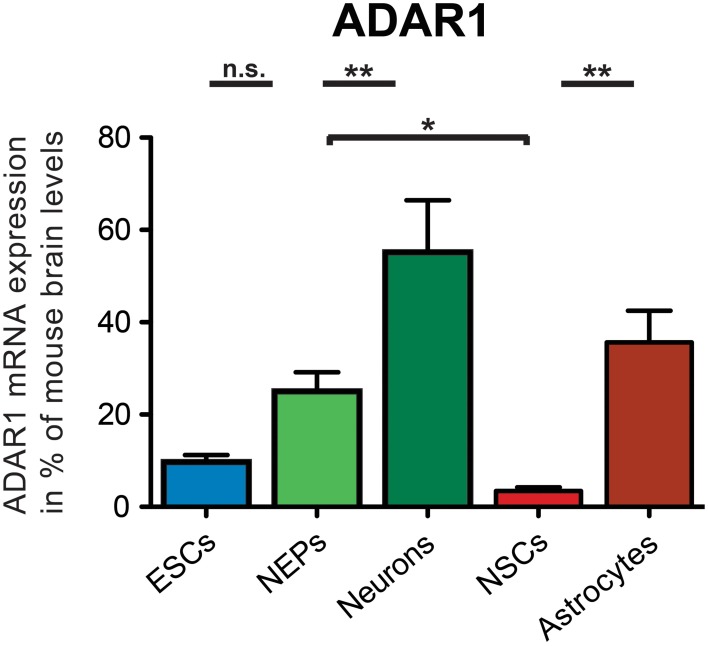
**Expression of ADAR1 mRNA in 46C cells**. The mRNA expression of ADAR1 was normalized to its expression in mouse whole brain P3 (2^ΔΔ*Ct*^). ADAR1 was expressed at the mRNA level in all investigated 46C-derived cell types, and its mRNA expression was upregulated significantly upon differentiation from 46C ESCs into NEPs and from 46C NEPs into neurons. By contrast, ADAR1 transcripts were significantly downregulated when 46C NEPs were differentiated into NSCs, and, subsequently, ADAR1 transcripts were significantly upregulated again following glial differentiation from 46C NSCs into astrocytes. Data represent means + SEM; statistical significances were assigned by One-Way ANOVA followed by Tukey's multiple comparison test; ^*^p < 0.05; ^**^p< 0.01; n = 4-8 independent experiments.

ADAR1 was expressed in all investigated 46C-derived cell types at the mRNA level (Figure 4). 46C ESCs and NEPs also already robustly expressed ADAR1 transcripts (9.7% and 25.02%, respectively, compared to expression in mouse whole brain). When 46C NEPs were differentiated into neurons, the mRNA expression of ADAR1 was significantly upregulated (*p* < 0.01; Figure 4), where it reached approximately half the expression level of the control tissue (55.2% compared to its expression in mouse whole brain). By contrast, when 46C NEPs were differentiated into NSCs, the expression of ADAR1 transcripts was downregulated (*p* < 0.05; Figure 4) and the mRNA expression of ADAR1 in 46C NSCs accounted for only 3.4% compared to its expression in mouse whole brain. Upon glial differentiation from NSCs into astrocytes, ADAR1 mRNA expression again increased significantly (*p* < 0.01; Figure 4) to 35.6% compared to its expression in mouse whole brain.

**Figure 5:**
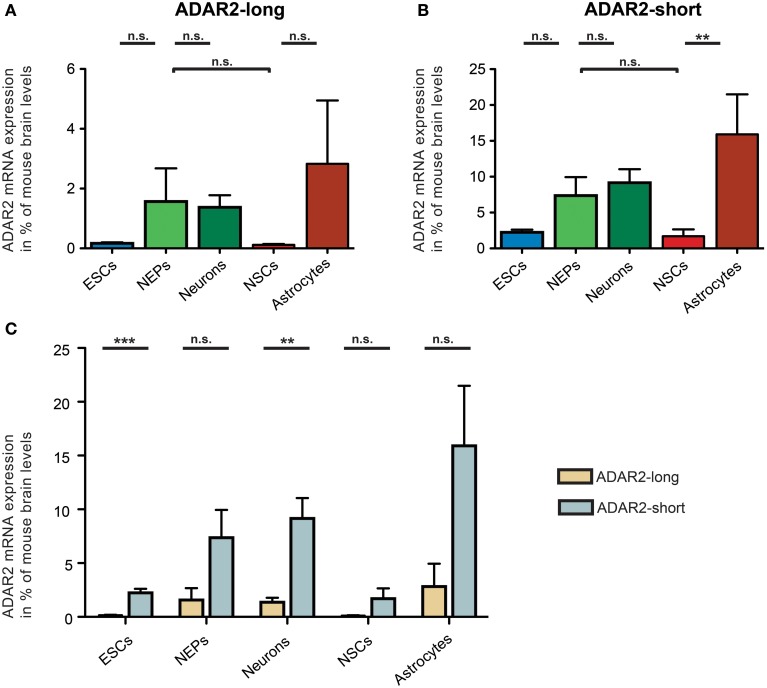
**Expression of ADAR2-long and ADAR2-short mRNAs in 46C cells**. The mRNA expression of ADAR2-long **(A)** and ADAR2-short **(B)** was normalized to its expression in mouse whole brain P3 (2^ΔΔ*Ct*^). ADAR2-long mRNA was expressed in all 46C cells, albeit only weakly in 46C ESCs and NSCs. Nevertheless, the mRNA expression did not differ significantly between the different 46C-derived cell types. ADAR2-short mRNA was expressed in all 46C cells as well, with mRNA expression levels not differing significantly between different 46C-derived cell types except following glial differentiation from NSCs into astrocytes. **(C)** Comparison of the expression of ADAR2-long and ADAR2-short mRNAs in 46C cells. The mRNA expression of ADAR2-short was higher in all 46C-derived cell types than that of ADAR2-long. This difference in expression was highly significant in 46C ESCs and neurons. Data represent means + SEM; statistical significances were assigned by One-Way ANOVA followed by Tukey's multiple comparison test; ^*^p < 0.01; ^***^p < 0.001; n.s. = not significant. n = 3-7 independent experiments.

Regarding ADAR2, the mRNA expression of two distinct ADAR2 splice variants was investigated (Figure 5). In 1997, it was shown that human ADAR2-short is twice as active as ADAR2-long in editing the Q/R site of GluA2 (Gerber et al., 1997). Similarly, rodent ADAR2 undergoes alternative splicing in the same exon as human ADAR2. Thus, the expression of both ADAR2-long and ADAR2-short in 46C cells was analyzed.

ADAR2-long was weakly expressed at the mRNA level in all investigated 46C-derived cell types, with expression levels not changing significantly upon differentiation from 46C ESCs into NEPs, or from 46C NEPs into NSCs, respectively. Moreover, the mRNA expression of ADAR2-long in 46C NEPs and neurons was roughly at the same level (1.6% compared to mouse whole brain for NEPs; 1.4% compared to mouse whole brain for neurons).

Likewise, ADAR2-short transcripts were also expressed in all investigated 46C-derived cell types. Its mRNA expression did not differ significantly between 46C ESCs and NEPs, or between 46C NEPs and NSCs, or between 46C NEPs and neurons. However, in contrast to ADAR2-long, the expression of ADAR2-short was significantly upregulated (*p* < 0.01; Figure 5B) upon glial differentiation from 46C NSCs into astrocytes (1.7% compared to mouse whole brain for NSCs; 15.9% compared to mouse whole brain for astrocytes).

In general, the mRNA expression of ADAR2-short was higher than that of ADAR2-long in all 46C-derived cell types (Figure 4C). In particular, ADAR2-short transcripts were significantly more strongly expressed in 46C ESCs (*p* < 0.001; Figure 4C) and neurons (*p* < 0.01; Figure 4C) than ADAR2-long transcripts. In 46C ESCs and in 46C neurons, ADAR2-short mRNA expression was 13-fold and 7-fold higher, respectively, than ADAR2-long mRNA expression.

**Figure 6:**
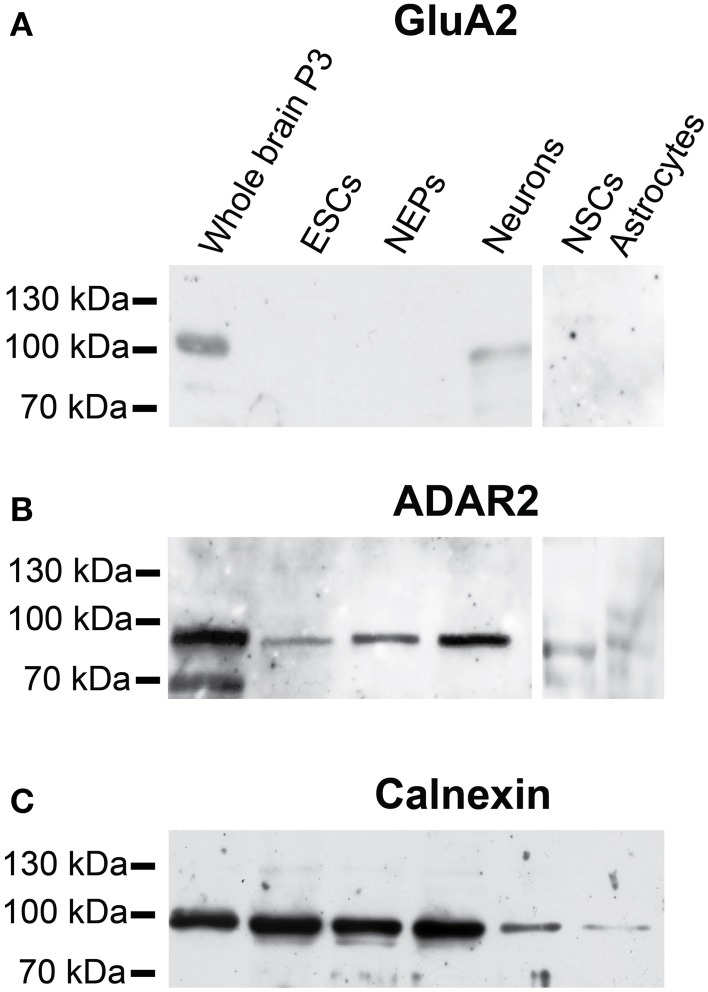
**Protein expression of GluA2 and ADAR2 in 46C cells**. **(A)** GluA2. **(B)** ADAR2. A band at the expected molecular weight of GluA2 (100 kDa) was only visible in the positive control (protein isolated from mouse whole brain P3) and in neurons differentiated from 46C NEPs. By contrast, ADAR2 (expected molecular weight: 90 kDa) was expressed in all 46C-derived cell types including undifferentiated ESCs. **(C)** Expression of calnexin as a loading control (90 kDa). n = 3 independent experiments.

Regarding the protein expression of GluA2 and ADAR2, GluA2 protein was only expressed in the positive control (membrane protein isolated from mouse whole brain P3) and in neurons differentiated from 46C NEPs (Figure 6A). As already described, neither 46C ESCs, NEPs, NSCs nor astrocytes differentiated from NSCs expressed GluA2 at the protein level (Pachernegg et al., 2013). By contrast, ADAR2 proteins were expressed in every 46C-derived cell type (Figure 6B) including undifferentiated 46C ESCs.

Thus, although GluA2 mRNA is not yet expressed in 46C ESCs, its editing enzyme ADAR2 is expressed in undifferentiated 46C ESCs at both the mRNA and protein levels.

### 3.2. Establishment of an editing assay

To determine the Q/R editing state of GluA2 in 46C cells, five different editing assay approaches were tested (Figure 2). Additionally, the R/G editing state of GluA2-GluA4 was investigated in a limited number of samples. The published *Bbv*I restriction assay to analyze the Q/R editing state of GluA2 was proven to be difficult, since the primers used in the semi-nested PCRs of GluA2 (Whitney et al., 2008) did not only amplify GluA2, but also GluA3. Thus, a quantitative analysis of the Q/R editing of GluA2 via the *Bbv*I restriction assay was not possible.

The four other editing assays differed in their cloning efficiencies (Table 2). The most efficient cloning approach was sticky end cloning, as 79% of the obtained sequences were indeed the desired AMPAR sequences. SLIC and blunt end cloning yielded roughly the same percentage of AMPAR sequences (46% and 43%, respectively). The least efficient cloning approach was the ligation with subsequent restriction; only 24% of the sequences obtained with this approach were AMPAR sequences. Consequently, most of the AMPAR sequences were obtained by sticky end cloning.

**Table 2:** **Efficiency of four different editing assays**.

**Cloning approach**	**Number of sequences**				
	**AMPAR**	**Different gene**	**Empty plasmid**	**Total**	**95% CI (AMPAR sequence)**
Sticky end	813 (79%)	185 (18%)	30 (3%)	1029	786 (76%)—837 (81%)
SLIC	63 (46%)	25 (18%)	48 (35%)	136	52 (38%)—74 (54%)
Blunt end	357 (43%)	155 (18%)	330 (39%)	842	329 (39%)—385 (46%)
Restriction after ligation	52 (24%)	48 (23%)	114 (53%)	214	41 (19%)—65 (30%)

Following different subcloning methods, the inserts of the plasmid DNAs of individual colonies were sequenced. Below, the results of four different approaches are listed, sorted by the percentage of AMPAR sequences obtained. Ninety five percent confidence intervals were calculated using the modified Wald method. AMPAR = AMPAR sequence successfully subcloned into pSGEM; Different gene = different gene sequence subcloned into pSGEM; Empty plasmid = re-ligated pSGEM; Total = total number of sequences obtained by each cloning approach; CI = confidence intervals.

**Table 3:** **Expression of GluA2, ADAR1, and ADAR2 mRNAs in 46C cells**.

**Cell type**	**GluA2**	**ADAR1**	**ADAR2-long**	**ADAR2-short**
	**mRNA expression levels (%)**		**normalized to housekeeping genes**	
46C ESCs	0	1.387 ± 0.216	0.039 ± 0.008	0.252 ± 0.042
46C NEPs	0.451 ± 0.176	3.565 ± 0.591	0.373 ± 0.264	0.897 ± 0.291
46C NSCs	0.1875 ± 0.086	0.489 ± 0.118	0.028 ± 0.006	0.191 ± 0.109
46C neurons	3.599 ± 0.725	7.864 ± 1.604	0.326 ± 0.097	1.031 ± 0.219
46C astrocytes	0.102 ± 0.022	5.074 ± 0.983	0.673 ± 0.505	1.791 ± 0.629
	**mRNA expression levels (%)**		**normalized to mouse whole brain**	
46C ESCs	0	9.7 ± 1.52	0.16 ± 0.04	2.2 ± 0.37
46C NEPs	7.8 ± 3.07	25.02 ± 4.17	1.6 ± 1.1	7.4 ± 2.6
46C NSCs	3.3 ± 1.54	3.4 ± 0.82	0.1 ± 0.02	1.7 ± 0.97
46C neurons	62.7 ± 12.64	55.2 ± 11.26	1.4 ± 0.4	9.1 ± 1.9
46C astrocytes	1.7 ± 0.39	35.6 ± 6.89	2.8 ± 2.1	15.9 ± 5.59

The mRNA expression of GluA2, ADAR1, ADAR2-long, and ADAR2-short was normalized to the expression of the housekeeping genes β-actin, GAPDH, and ubiquitin (2^Δ*Ct*^) and to their expression in mouse whole brain (2^ΔΔ*Ct*^). Data represent means ± SEM; n = 3-15 independent experiments.

### 3.3. Q/R editing state of GluA2 in 46C cells

The Q/R editing state of GluA2 was investigated in all 46C-derived cell types, namely ESCs, NEPs, NSCs, neurons, and astrocytes, as well as in control tissue (mouse whole brain P3) (Table 4). Approximately 10–15% of the sequences in a given cell type were GluA3 sequences, since the semi-nested primers for GluA2 also amplified GluA3. 46C ESCs did not express GluA2 at all, thus, all sequences obtained in this cell type were GluA3 sequences. In all other 46C-derived cell types and in mouse whole brain, GluA2 mRNA was expressed and 102–113 individual GluA2 sequences were analyzed per cell type. In the control tissue, 100% of GluA2 subunits were edited at the Q/R site. The same holds true for 46C NEPs, neurons, and astrocytes. In 46C NSCs, 106 out of 107 GluA2 sequences were edited at the Q/R site, yielding 99% Q/R-edited GluA2 subunits in NSCs.

**Table 4:** **Q/R editing state of GluA2 in 46C cells**.

**Cell/tissue type**	**Number of sequences**			
	**Edited GluA2/Total GluA2**	**GluA3**	**Total**	**95% Confidence intervals (edited GluA2)**
46C ESCs	n.a.	26	26	n.a.
46C NEPs	113/113 (100%)	11	124	108 (95.6%)—113 (100%)
46C NSCs	106/107 (99.1%)	10	117	101 (94.4%)—107 (100%)
46C neurons	105/105 (100%)	12	117	100 (95.2%)—105 (100%)
46C astrocytes	105/105 (100%)	10	115	100 (95.2%)—105 (100%)
mouse whole brain P3	102/102 (100%)	11	113	97 (95.1%)—102 (100%)

The results obtained by sequencing GluA2 from individual bacterial colonies are given below. Additionally, the numbers of GluA3 sequences unintentionally co-amplified in the RT-PCR are listed. Ninety five percent confidence intervals were calculated using the modified Wald method. n.a. = no sequences obtained due to lack of expression; Total = total number of sequences obtained for each cell/tissue type.

### 3.4. Q/R editing state of GluA2 during differentiation from 46C ESCs to NEPs

Since 46C ESCs did not express GluA2 at all and in 46 NEPs, 100% of GluA2 was already edited at the Q/R site, the question arose whether there are any Q/R-unedited GluA2 subunits during the differentiation from 46C ESCs to NEPs. The differentiation from 46C ESCs to NEPs takes 9 days (Aubert et al., 2003; Ying et al., 2003; Muth-Köhne et al., 2010a,b) and total RNA was isolated from the differentiating 46C cells at various time points (d0, d2, d4, d4.5, d5, d7, and d9 of differentiation).

The mRNA expression of GluA2 started after 4.5 days of differentiation of 46C ESCs into NEPs. Prior to that time point, no bands were visible in the agarose gel electrophoresis following the RT and semi-nested PCR, and when the PCR reaction products were directly cloned into the pSGEM vector, only contaminating co-amplified GluA3 sequences were obtained (Table 5). After 4.5, 5, 7, and 9 days of differentiation from 46C ESCs into NEPs, GluA2 sequences were obtained. However, only after 4.5 days of differentiation Q/R-unedited GluA2 subunits were identified (9 out of 102 total GluA2 sequences = 8.8% Q/R-unedited GluA2). After 5, 7, and 9 days of differentiation, 100% of GluA2 was edited at the Q/R site.

**Table 5:** **Q/R editing state of GluA2 during the differentiation from 46C ESCs into NEPs**.

**Time point of differentiation**	**Number of sequences**			
	**Edited GluA2/Total GluA2**	**GluA3**	**Total**	**95% Confidence intervals (edited GluA2)**
d0 (46C ESCs)	n.a.	9	9	n.a.
d2	n.a.	11	11	n.a.
d4	n.a.	15	15	n.a.
d4.5	93/102 (91.1%)	11	113	85 (83.3%)—97 (95.1%)
d5	108/108 (100%)	12	120	103 (95.4%)—108 (100%)
d7	112/112 (100%)	10	122	107 (95.5%)—112 (100%)
d9 (46C NEPs)	107/107 (100%)	11	118	102 (95.3%)—107 (100%)

The results obtained by sequencing GluA2 are given below. Additionally, the numbers of GluA3 sequences unintentionally co-amplified in the RT-PCR are listed. Ninety five percent confidence intervals were calculated using the modified Wald method. d = day of differentiation; n.a. = no sequences obtained due to lack of expression; Total = total number of sequences obtained for each time point.

### 3.5. R/G editing state and expression of flip/flop splice variants of AMPARs in 46C cells

Although this study focused on the Q/R editing state of GluA2, the R/G editing state of GluA2, GluA3, and GluA4 and the expression of different AMPAR splice variants in 46C cells was briefly investigated as well. To this end, specific primers were used in the PCRs that amplify fragments of AMPARs that contain both the R/G editing site and the flip/flop splicing region. Again, total RNA isolated from all types of 46C-derived cells (ESCs, NEPs, NSCs, neurons, and astrocytes) was used.

The total number of sequences obtained in the R/G editing assay was low. Nevertheless, the data clearly show that all cell types expressed both R/G-edited and -unedited AMPARs (Table 6), as well as both the flip and flop splice variant. This is in stark contrast to Q/R editing, in which Q/R-unedited GluA2 subunits were substantially expressed only in a very brief time window during the differentiation from 46C ESCs to NEPs.

**Table 6:** **Flip/flop splice variant and R/G editing state of AMPARs in 46C cells**.

**AMPAR**	**Splice variant**		**R/G editing site**		**Total**	**95% CI (Flip)**	**95% CI (R/G-unedited)**
	**Flip**	**Flop**	**Unedited**	**Edited**			
**46C ESCs**							
GluA1	6 (60%)	4 (40%)	–	–	10	3 (30%)—8 (80%)	–
GluA2	n.a.	n.a.	n.a.	n.a.	n.a.	n.a.	n.a.
GluA3	12 (92%)	1 (8%)	9 (69%)	4 (31%)	13	8 (61%)—13 (100%)	5 (38%)—11 (85%)
GluA4	8 (100%)	0 (0%)	7 (88%)	1 (12%)	8	5 (62%)—8 (100%)	4 (50%)—8 (100%)
**46C NEPs**							
GluA1	5 (83%)	1 (17%)	–	–	6	2 (33%)—6 (100%)	–
GluA2	7 (78%)	2 (22%)	9 (100%)	0 (0%)	9	4 (44%)—8 (89%)	6 (67%)—9 (100%)
GluA3	4 (80%)	1 (20%)	3 (60%)	2 (40%)	5	2 (40%)—5 (100%)	1 (20%)—4 (80%)
GluA4	6 (100%)	0 (0%)	6 (100%)	0 (0%)	6	3 (50%)—6 (100%)	3 (50%)—6 (100%)
**46C NSCs**							
GluA1	0 (0%)	7 (100%)	–	–	7	0 (0%)—3 (43%)	–
GluA2	6 (100%)	0 (0%)	1 (17%)	5 (83%)	6	3 (50%)—6 (100%)	0.06 (1%)—3 (50%)
GluA3	12 (100%)	0 (0%)	0 (0%)	12 (100%)	12	8 (67%)—12 (100%)	0 (0%)—3 (25%)
GluA4	1 (100%)	0 (0%)	0 (0%)	1 (100%)	1	0.16 (16%)—1 (100%)	0 (0%)—0.83 (83%)
**46C neurons**							
GluA1	7 (100%)	0 (0%)	–	–	7	4 (57%)—7 (100%)	–
GluA2	9 (100%)	0 (0%)	7 (78%)	2 (22%)	9	6 (67%)—9 (100%)	4 (44%)—8 (89%)
GluA3	4 (100%)	0 (0%)	2 (50%)	2 (50%)	4	2 (50%)—4 (100%)	0.6 (15%)—3 (75%)
GluA4	8 (89%)	1 (11%)	8 (89%)	1 (11%)	9	8 (89%)—9 (100%)	8 (89%)—9 (100%)
**46C astrocytes**							
GluA1	6 (86%)	1 (14%)	–	–	7	3 (43%)—7 (100%)	–
GluA2	8 (89%)	1 (11%)	0 (0%)	9 (100%)	9	5 (55%)—9 (100%)	0 (0%)—3 (33%)
GluA3	6 (100%)	0 (0%)	2 (33%)	4 (67%)	6	3 (50%)—6 (100%)	0.5 (8%)—4 (67%)
GluA4	11 (100%)	0 (0%)	0 (0%)	11 (100%)	11	8 (73%)—11 (100%)	0 (0%)—3 (27%)

The results obtained by sequencing PCR-amplified and subcloned AMPAR fragments are given below. Ninety five percent confidence intervals were calculated using the modified Wald method. n.a. = no sequences obtained due to lack of AMPAR expression; − = lack of R/G editing in GluA1; Total = total number of sequences obtained for each subunit; CI = confidence intervals.

## 4. Discussion

### 4.1. ADARs are expressed in 46C ESCs prior to the onset of GluA2 expression

In the present study, the mRNA expression of GluA2 and its editing enzymes ADAR1 and ADAR2, as well as the protein expression of ADAR2 and GluA2 were analyzed. ADAR1 protein expression was not investigated, since its editing efficiency at the Q/R site of GluA2 is relatively low compared to ADAR2 (Liu and Samuel, 1999; Wong et al., 2001).

Regarding the mRNA expression of GluA2, it was not yet expressed in 46C ESCs but its transcript expression started only in NEPs, in which GluA2 was not expressed at the protein level (see also Pachernegg et al., 2013). The mRNA expression of GluA2 was then significantly upregulated upon neuronal differentiation, and 46C neurons also expressed GluA2 protein.

**Figure 7:**
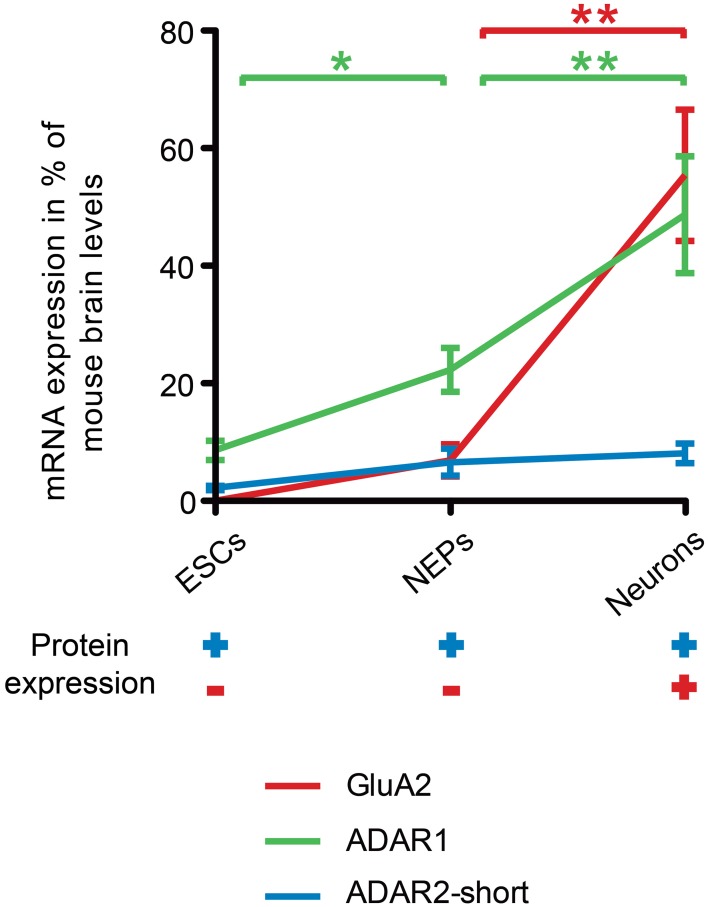
**Comparison of ADAR, GluA2 mRNA and protein expression time lines during neural differentiation of 46C ESCs**. The expression of ADAR2-short (blue line) precedes the expression of GluA2 (red line) at both the mRNA and protein levels. ADAR2-short mRNA expression was roughly the same in 46C ESCs, NEPs, and neurons, whereas the transcript expression of both ADAR1 and GluA2 increased significantly upon neuronal differentiation. Moreover, the mRNA expression of ADAR1 (green line) was also significantly higher in 46C NEPs than in ESCs. The upregulation of ADAR1 may be related to the gradual increase of R/G editing during neural development. Furthermore, ADAR2-short was already expressed at the protein level in undifferentiated 46C ESCs and NEPs, whereas GluA2 proteins were only detectable after neuronal differentiation. Note that the mRNA expression of ADAR2-long was not depicted here for simplification. Data represent means ± SEM; statistical significances were assigned by One-Way ANOVA followed by Tukey's multiple comparison test; ^*^p < 0.05; ^**^p < 0.01; n = 3-15 independent experiments.

By contrast, both ADAR1 and ADAR2 transcripts were already expressed in 46C ESCs, and, for that matter, in all 46C-derived cell types. Surprisingly, ADAR2 protein was also expressed in all investigated 46C-derived cell types, even in ESCs and NEPs, although GluA2 protein was not expressed until differentiation into neurons. Hence, ADAR2 expression precedes the expression of GluA2 at both the mRNA and protein levels (Figure 7). Furthermore, the mRNA expression of ADAR2 was roughly identical in 46C ESCs, NEPs, and neurons, and did not change significantly upon neuronal differentiation, indicating that the relatively low expression of ADAR2 in comparison to ADAR1 is sufficient to ensure efficient Q/R editing. This matches the reported widespread, yet weak, mRNA, and protein expression of ADAR2 in mouse embryonic forebrain around E15 (Jacobs et al., 2009). Nevertheless, GluA2 is almost completely edited at the Q/R site at this time point (Jacobs et al., 2009). Thus, the authors speculate that ADAR2 preferentially edits the Q/R site of GluA2, and that a low ADAR2 expression is sufficient for Q/R editing of all GluA2 transcripts present (Jacobs et al., 2009).

Additionally, the overall transcript expression of ADAR2-short was higher than the transcript expression of ADAR2-long in all 46C cell types. Human ADAR2-short is twice as active as ADAR2-long in editing the Q/R site of GluA2 (Gerber et al., 1997). The predominant expression of ADAR2-short compared to ADAR2-long also hints at the establishment of a pool of ADAR2 in 46C ESCs and NEPs to rapidly edit GluA2 at the Q/R site.

By contrast, the mRNA expression of ADAR1, which is mainly involved in the developmentally gradually increasing R/G editing, significantly increases during differentiation of 46C-derived cells. Furthermore, ADAR1 mRNAs are more abundantly expressed than ADAR2 transcripts. In general, ADAR1 seems to have a higher impact on the development of the haematopoietic system than on neural development: ADAR1 knockout (KO) mice die early during embryonic development due to failures in the haematopoietic system (Wong and Lazinski, 2002), but they do not show abnormalities related to brain development. Furthermore, ADAR1 was shown to be essential for the survival of haematopoietic progenitor cells (XuFeng et al., 2009). It might be that 46C-derived cells express ADAR1 targets apart from AMPARs and that editing of these targets might be crucial for the survival of ESCs. However, the target gene(s) of ADAR1 which cause the lethal phenotype of ADAR1 KO mice remain unknown thus far (Riedmann et al., 2008).

### 4.2. Establishment of an editing assay

To analyze the Q/R editing state of GluA2 in 46C cells, five different editing assays were tested: *Bbv*I restriction digest and four sequencing-based assays relying on blunt end cloning, ligation with subsequent restriction, sticky end cloning, or SLIC cloning.

The *Bbv*I restriction digest assay (Whitney et al., 2008) is based on the fact that the 271 bp long GluA2 fragment amplified by semi-nested PCRs contains two restriction sites for *Bbv*I when the Q/R site is unedited and only one *Bbv*I site when it is edited. Thus, Q/R-unedited and -edited GluA2 subunits yield different restriction digest patterns upon restriction with *Bbv*I. However, approximately 10–15% of the DNA fragments represented contaminating GluA3 rather than GluA2 sequences, as revealed by DNA sequencing performed during the other editing assays. Given the high sequence identity of AMPARs and only minor differences in the published primer binding sites between GluA2 and GluA3 (1 and 2 bases for the sense and antisense primers, respectively) it is comprehensible that GluA3 was easily co-amplified in the semi-nested PCRs. This is of particular importance for 46C ESCs (and other cells), in which GluA3 but not yet GluA2 is expressed at the mRNA level. The unintended co-amplification of GluA3 will distort the results in the *Bbv*I restriction assay, since GluA3 contains two restriction sites for *Bbv*I as well. GluA3 is never edited at the Q/R site, and a restriction digest of the amplified GluA3 fragment with *Bbv*I will always yield three DNA fragments (151 bp, 81 bp, and 45 bp) that in practice are indistinguishable from DNA fragments obtained by the restriction of Q/R-unedited GluA2 (145 bp, 80 bp, and 46 bp). Thus, the *Bbv*I restriction assay is not suitable for investigating the Q/R editing state of GluA2, because the unavoidable contamination by GluA3 inevitably leads to overestimation of the fraction of Q/R-unedited GluA2. Rather, an approach including the sequencing of DNA fragments should be chosen that allows to sort out the contaminating GluA3 sequences.

The other four editing assays were all based on the subcloning of PCR fragments into the pSGEM vector, its transformation into bacteria, and the subsequent preparation and sequencing of plasmid DNA. By doing so, not only the co-amplification of GluA3 could be monitored, but by subcloning the PCR fragments into bacteria a high number of sequences for a reliable statistical evaluation could be obtained as well, since this method allows to sequence the plasmid DNA of many individual bacterial colonies.

Ultimately, the four sequencing-based editing assays differed only in the way the PCR fragments were subcloned into the pSGEM vector. In this regard, the best approach turned out to be sticky end cloning, which yielded the highest percentage (approximately 80%) of AMPAR-specific sequences. For the editing assay, as many informative plasmid/insert constructs as possible should be obtained to increase the number of total sequences and thus improve the statistics. In order to do so, most GluA2 sequences were thus obtained by sticky end cloning in this study.

### 4.3. GluA2 is rapidly edited at the Q/R site during differentiation from 46C ESCs to NEPs

After establishing a reliable editing assay, the Q/R editing state of GluA2 in 46C-derived cells was determined. As mentioned above, GluA2 mRNA was not yet expressed in undifferentiated 46C ESCs, but in NEPs. In 46C NEPs, 100% of GluA2 was already edited at the Q/R site, and, likewise, GluA2 was completely Q/R-edited in neurons and astrocytes. Only in 46C NSCs, a single Q/R-unedited GluA2 sequence (out of 107 sequences in total) was identified.

Furthermore, the Q/R editing state of GluA2 was analyzed in more detail at various time points during the differentiation of 46C ESCs into NEPs, which takes 9 days to complete (Aubert et al., 2003; Ying et al., 2003; Muth-Köhne et al., 2010a,b). GluA2 transcript expression set in after 4.5 days of differentiation of 46C ESCs into NEPs, and at this time point, less than 10% of GluA2 subunits were unedited at the Q/R site. Only 0.5 days later, at day 5 of differentiation, GluA2 was already completely Q/R-edited.

Although the exact onset of Q/R editing during neural development *in vivo* is still a matter of debate, Q/R editing undoubtedly is more rapid and thorough than R/G editing, which increases only gradually and never reaches 100%, not even in adults (Lomeli et al., 1994; Geiger et al., 1995; Lai et al., 1997; Orlandi et al., 2011). It is possible that a pool of ADAR2 is established in ESCs and NEPs to ensure the rapid Q/R editing of GluA2 as soon as the mRNA expression of GluA2 sets in in NEPs. Moreover, when GluA2 is finally expressed at the protein level in neurons, the vast majority, if not all, GluA2 subunits will be edited at the Q/R site. Our data is in line with previous studies, which showed that GluA2 is already almost completely Q/R-edited in the embryonic and postnatal brain (Longone et al., 1998; Kawahara et al., 2004; Jacobs et al., 2009; Wahlstedt et al., 2009; Shtrichman et al., 2012).

It is important to note that ADAR2 expression and GluA2 Q/R editing are not linearly related. The editing assay in this study does not detect Q/R-unedited GluA2 pre-mRNA, since the primers used in this assay span at least one exon-exon junction. Thus, putative Q/R-unedited GluA2 pre-mRNA was not detected in this study and could be present both in ESCs and NEPs. However, the basal expression of ADAR2 in ESCs and its increase of expression after differentiation into NEPs argue against a substantial amount of Q/R-unedited GluA2 pre-mRNA in these cells.

The importance of ADAR2 and Q/R editing was proven by the generation of KO animals. Homozygous ADAR2^−/−^ mice die between P0 and P21 and are subject to seizures, presumably caused by the 30-fold higher Ca^2+^ permeability and higher single channel conductance of AMPARs with Q/R-unedited GluA2 subunits in CA1 pyramidal neurons (Higuchi et al., 2000). Interestingly, the severe effect of ADAR2 KO can be rescued by introducing a “pre-edited” GluA2 gene. Moreover, heterozygous ADAR2^−/−^/GluA2^+/*R*^ mice, in which only one allele of GluA2 expresses GluA2(R), are also partially rescued: Their phenotype is less impaired than that of ADAR2^−/−^ mice and they survive until P35 (Higuchi et al., 2000). Homozygous ADAR2^−/−^/GluA2^*R*/*R*^ mice, in which both alleles of GluA2 express GluA2(R), are fully rescued and show no abnormality in postnatal development or general behavior (Higuchi et al., 2000). Thus, it appears that GluA2 is the most important substrate for ADAR2 in terms of viability. In addition, heterozygous GluA2^Δ*ECS*^ mice, in which the Q/R editing of GluA2 is abolished due to a mutation in the editing-determining exon complementary sequence (ECS), die at P20 and show an early onset of epilepsy like ADAR2^−/−^ mice (Brusa et al., 1995; Feldmeyer et al., 1999). Altogether, these findings point to a crucial role of Q/R editing of GluA2 during embryonic and neonatal development. Thus, it is unlikely that the early onset of ADAR2 expression in 46C cells is (substantially) linked to the editing of a target mRNA other than GluA2.

In contrast to our data, Whitney et al. (2008) found approximately 60% of Q/R-unedited GluA2 subunits in human neural progenitor cells. The diverging results might be caused by the differences between human and murine cells. However, Whitney et al. (2008) had used the *Bbv*I restriction assay, which is not well-suited to detect Q/R-unedited GluA2 subunits, since it also cross-detects GluA3 subunits that are never edited at the Q/R site. In the present study, in every investigated cell type (and in the control tissue mouse whole brain) approximately 10–15% of obtained sequences were GluA3 sequences, which would have been regarded as “Q/R-unedited GluA2” subunits with the *Bbv*I restriction assay. Thus, the extent of Q/R-unedited GluA2 subunits in human neural progenitor cells is probably substantially lower than reported, if there are any at all. Furthermore, there is increasing evidence that Ca^2+^-permeable AMPARs lacking GluA2 subunits play an important role in NMDAR-independent LTP triggering (Jia et al., 1996; Harvey et al., 2001; Meng et al., 2003; Wiltgen et al., 2010). However, Ca^2+^-permeable AMPARs that contain Q/R-unedited GluA2 subunits have not been identified in the healthy brain thus far, but only following ischemia (Peng et al., 2006) or in motor neurons of ALS patients (Kwak and Kawahara, 2005). Thus, while Whitney et al. (2008) found Ca^2+^-permeable AMPARs in human neural progenitor cells, it is likely that a majority of these receptors were composed of GluA1, GluA3, or GluA4 subunits, but did not contain Q/R-unedited GluA2 subunits. Furthermore, GluA2^*R*/*R*^ mice, which are genetically edited at the Q/R site, are viable and show no differences in brain architecture (Kask et al., 1998). Our findings support the hypothesis that Q/R-unedited GluA2 does not play a role during neural development (Kask et al., 1998), but is rather rapidly edited at the Q/R site as soon as GluA2 mRNA expression sets in. Moreover, the severe effects of ADAR2 KO, which can be rescued by “pre-edited” GluA2, argue against a significant expression of Q/R-unedited GluA2 subunits during neural development (Higuchi et al., 2000).

### 4.4. AMPARs are not fully edited at the R/G site in 46C cells

Although this study focused on the Q/R editing state of GluA2, for which the bulk of the data has been collected, it is obvious from the data obtained for the R/G editing site that AMPARs are not as rapidly and efficiently edited at the R/G site during neural development as GluA2 is at the Q/R site. In all investigated 46C cell types, R/G-unedited subunits are expressed at the mRNA level. Moreover, AMPARs are not expressed at the protein level in 46C ESCs or NEPs (Pachernegg et al., 2013), and once AMPAR protein expression sets in after differentiation into neurons, both R/G-edited and -unedited AMPARs can be found expressed. This is consistent with previous studies that reported a gradual increase of R/G editing during neural development (Lomeli et al., 1994; Geiger et al., 1995; Lai et al., 1997; Orlandi et al., 2011). Hence, the 46C cell system is well-suited to investigate the editing state of AMPARs during neural development since it faithfully mimics the conditions *in vivo*.

### 4.5. Conclusions

This study analyzed for the first time the precise onset of GluA2 mRNA expression during differentiation of ESCs into NEPs, and, additionally, the exact onset of Q/R editing during this time course. GluA2 subunits are rapidly edited during neural differentiation, and Q/R-unedited GluA2 subunits are only present within a very brief time window. The preceding expression of ADAR2 both at the mRNA and protein levels argues for the presence of a pool of ADAR2 in undifferentiated ESCs to ensure the rapid Q/R editing of GluA2. These results partly contradict literature data. However, in the present study, the Q/R editing state of GluA2 was determined with a more reliable editing assay than in previous studies. In future studies, the physiological consequences of the rapid onset of Q/R editing should be investigated.

## Author contributions

SP designed the study, carried out the experiments, and drafted the manuscript. YM and GF helped in collecting data. EMK participated in cell culture work and in designing the study. MH participated in the design and supervision of the study. All authors read and approved the final manuscript.

### Conflict of interest statement

The authors declare that the research was conducted in the absence of any commercial or financial relationships that could be construed as a potential conflict of interest.
